# Dimethyl fumarate abrogates dust mite‐induced allergic asthma by altering dendritic cell function

**DOI:** 10.1002/iid3.262

**Published:** 2019-07-02

**Authors:** Anil K. Jaiswal, Maninder Sandey, Amol Suryawanshi, Russell C. Cattley, Amarjit Mishra

**Affiliations:** ^1^ The Laboratory of Lung Inflammation, College of Veterinary Medicine Auburn University Auburn Alabama; ^2^ Department of Pathobiology, College of Veterinary Medicine Auburn University Auburn Alabama

**Keywords:** allergic asthma, dendritic cells, dimethyl fumarate, house dust mite

## Abstract

**Introduction:**

Allergic asthma is the most common inflammatory disease of upper airways. Airway dendritic cells (DCs) are key antigen presenting cells that regulate T helper 2 (Th2)‐dependent allergic inflammation. Recent studies have shown critical role of airway DCs in the induction of Th2‐mediated allergic inflammation and are attractive therapeutic targets in asthma. However, molecular signaling mechanism that regulate DCs function to Th2 immune responses are poorly understood. Here we aim to evaluate the immunomodulatory effect of dimethyl fumarate (DMF), an FDA approved small molecule drug, in the house dust mite (HDM)‐induced experimental model of allergic asthma.

**Methods:**

DMF was administered intranasally in the challenge period of HDM‐induced murine model of experimental asthma. Airway inflammation, airway hyperreactivity, Th2/Th1 cytokine were assessed. The effect of DMF on DC function was further evaluated by adoptive transfer of HDM‐pulsed DMF treated DCs to wild‐type naïve mice.

**Results:**

DMF treatment significantly reduced HDM‐induced airway inflammation, mucous cell metaplasia, and airway hyperactivity to inhaled methacholine. Mechanistically, DMF interferes with the migration of lung DCs to draining mediastinal lymph nodes, thereby attenuates the induction of allergic sensitization and Th2 immune response. Notably, adoptive transfer of DMF treated DCs to naïve mice with HDM challenge similarly reduces the features of allergic asthma.

**Conclusion:**

This identifies a novel function of DMF on DC‐mediated adaptive immune responses in the setting of HDM‐induced airway inflammation. Taken together, our results offer a mechanistic rationale for DMF use to target DCs in local lung environment as antiasthmatic therapy.

**Abbreviations**
AF: Alexa Fluor
AHR: airway hyperresponsiveness
BAL: bronchoalveolar lavage
BMDC: bone marrow–derived dendritic cells
DCs: dendritic cells
DMF: dimethyl fumarate
Foxp 3: forkhead box protein 3
mLN: mediastinal lymph node
HDM: house dust mite
Tregs: regulatory T cells

## 1 INTRODUCTION

Dimethyl fumarate (DMF), an α, β‐unsaturated carboxylic acid ester is a key derivative of the Krebs cycle intermediate fumarate and modulates immune cell functions, whereby effective in the treatment of immune‐mediated diseases.^1^, ^2^, ^3^ DMF is an attractive small molecule drug that is approved by the FDA and European Medicines for the treatment of autoimmune diseases such as, psoriasis and multiple sclerosis (MS).^4^, ^5^, ^6^ In immune cells, the fumaric acid ester DMF and its biologically active metabolite monomethyl fumarate (MMF) inhibit the rate‐limiting GAPDH enzyme activity by covalently binding with the cysteine residues, thereby block aerobic glycolysis and averts immune cell activation.^7^, ^8^, ^9^ This represents per se an important mechanism of DMF action, which reprogram the metabolic fate and skew immune cells towards inflammatory or regulatory functions.^10^

The electrophilic fumarate ester DMF exerts its antioxidant effect by covalently modifying nucleophile cysteine residues (thiol groups) of macromolecules such as, kelch‐like ECH‐associated protein 1 (KEAP1), which are known to activate nuclear‐factor (erythroid‐derived 2)‐related factor 2 (Nrf 2)‐dependent antioxidant response element (ARE) pathway.^11^, ^12^, ^13^ DMF‐mediated ARE‐dependent transcriptional induction promotes gene expression of the glutathione S‐transferase A2 (GSTA 2), hemeoxygenase (HO), and quinone oxidoreductase 1 (NQO1) enzymes, which are essential for cellular detoxification.^14^, ^15^ DMF effectively depletes intracellular glutathione (GSH) storage and induces interleukin 10 (IL‐10) producing dendritic cells (DCs) and T helper 2 (Th2) differentiation, which protects mice from experimental autoimmune encephalomyelitis (EAE) and psoriasis.^16^, ^17^ However, this anti‐inflammatory effect of DMF is independent of Nrf2 pathway.^10^ Although, oral DMF treatment is safe for patients with relapsing‐remitting MS, psoriasis and other inflammatory diseases, early stage adverse effect of flushing and GI tract events has been reported with several clinical trials.^4^, ^18^ Long‐term systemic DMF treatment have been shown to impact circulating immune cells and is associated with lymphopenia and progressive multiple leukoencephalopathy (PML).^19^, ^20^ Perhaps more importantly, DMF treatment alters T cell subset in peripheral blood and facilitates T cell polarization toward an anti‐inflammatory state, thereby correlates with the clinical improvement of MS patients.^21^, ^22^

Airway DCs are essential to sample inhaled antigens, consequently initiate, perpetuate and propagate allergic immune responses in asthma.^23^, ^24^, ^25^ The importance of airway DCs has been attributed as conditional removal of DCs reduced all the cardinal manifestations of asthma, including Th2 cytokine‐driven eosinophilic airway inflammation, mucous cell metaplasia, and airway hyperreactivity.^26^ Of interest, the compounds that modulate DCs function in airways and interfere with their migration process to draining lymph nodes, likely to be effective as novel antiasthmatic drugs. However, to avoid systemic effects, site‐directed delivery of these compounds directly to the lungs via inhalation would represent the preferred method of administration. The precise action of DMF on lung DCs function and Th2 adaptive immune regulation remains unknown.

Since DMF application can influence myriad of therapeutic targets and modify the balance of inflammatory and regulatory immune cell types, we hypothesized that it might also modulate airway DCs function and adaptive Th2‐mediated immune responses to house dust mite (HDM) antigen. We also hypothesized that if local DMF administration during HDM challenged phase could abrogate the cardinal features of HDM‐induced allergic asthma. Our current study shows that local administration of DMF reduced airway inflammation, mucous cell metaplasia and airway hyperactivity as well as impedes migration of DCs to draining mediastinal lymph node. Furthermore, we show that DMF interferes with DCs‐driven allergic sensitization and Th2 adaptive immune responses. This demonstrates a previously unidentified consequence of DMF treatment and support the concepts of locally acting compound as to modulate the regulatory function of airway DCs in allergic asthma.

## 2 MATERIALS AND METHODS

### 2.1 Quantitative reverse transcription polymerase chain reaction

RNA was isolated from the splenic DCs following HDM stimulation with or without DMF treatment using Trizol reagent (Life Technologies, Grand Island, NY) and complementary DNA (cDNA) was generated using a High Capacity RNA‐to‐cDNA kit (Applied Biosystems). The cDNA was pre‐amplified using previously described primers (Table 1). Ten‐microliter polymerase chain reaction (PCR) reactions were set up containing 0.1 µL of template DNA at a concentration of 20 ng/mL, 5 µL of PowerUP SYBR Green master mix (Applied Biosystems), 0.5 µL of each primer at a concentration of 20 µM and 3.9 µL of nuclease‐free water. Quantitative PCR was performed on the QuantStudio 7 Flex (Applied Biosystems) using the following conditions: one cycle at 50°C for 2 minutes, one cycle at 95°C for 2 minutes and then 40 cycles at 95°C for 15 seconds and 60°C for 1 minute, followed by a dissociation stage at 95°C for 15 seconds (1.6°C/s), 60°C for 1 minute (1.6°C/s) and 95°C for 15 seconds (0.15°C/s). After amplification, *C*q values were obtained and analyzed using DataAssist software (Applied Biosystems).

**Table 1:** Real‐time polymerase chain reaction primers

Genes	Forward (5′‐3′)	Reverse (3′‐5′)
IL 10	AGCCGGGAAGACAATAACTGC	CTGCATTAAGGAGTCGGTTAG
ICOS‐L	AGCTTGAACTTACAGACCACGC	CTCTGAAGTTGTGTCTGACATC
ST2	TGACGGCCACCAGATCATTCACAG	GCCAAAGCAAGCTGAACAGGCAATAC
OX40‐L	ATGGAAGGGGAAGGGGTTCAACC	TCACAGTGGTACTTGGTTCACAG
Batf3	CAGACCCAGAAGGCTGACAAG	CTGCGCAGCACAGAGTTCTC
Zbtb46	AGAGAGCACATGAAGCGACA	CTGGCTGCAGACATGAACAC
Irf 4	ACAGCACCTTATGGCTCTCTG	ATGGGGTGGCATCAT GTAGT
GAPDH	CCTGCACCACCAACTGCTTAG	GTGGATGCAGGGATGATGTTC

### 2.2 Reagents

DMF was from Selleckchem (Houston, TX) and HDM (*Dermatophagoides pteronyssinus*) extract was purchased from Greer Laboratories (Lenoir, NC) as a freeze‐dried preparation (item no. B82). Quantitative enzyme linked immunosorbent assay kits for measurements of CC‐chemokine ligands were from R&D Systems (Minneapolis, MN). Recombinant mouse granulocyte‐macrophage colony‐stimulating factor was from BioLegend (San Diego, CA) and recombinant mouse IL‐4 was from Life technologies Corporation (Grand Island, NY).

### 2.3 Mice

Six‐ to eight‐week‐old female mice were utilized for experiments and were purchased from The Jackson Laboratories (Bar Harbor, MA). Murine experimental protocols were approved by the Animal Care and Use Committee of the Auburn University (Auburn, AL).

### 2.4 HDM sensitization and challenge models

(a) Female Balb/c mice (6‐8 weeks old) were sensitized by intraperitoneal injection of HDM (100 µg) emulsified in 200 µL of phosphate‐buffered saline (PBS) containing 3 mg of aluminum hydroxide (Sigma‐Aldrich) on days 0 and 4. Mice were challenged by intranasal (i.n.) administration of HDM (100 µg) in a volume of 40 µL on days 8, 11, and 12 and end points were analyzed on day 14. Mice were intranasally (i.n.) administered with vehicle (2.8% dimethyl sulfoxide in PBS) or DMF (0.5 mg/kg bwt) in a total volume of 40 µL, 30 minutes before HDM challenge in the allergen challenge phase of the experimental asthma protocol. (b) Adoptive transfer of CD11c^+^ BMDCs treated with vehicle and DMF. Bone marrow cells were isolated from the leg bones of euthanized Balb/c mice and cultured in T‐25 cm^2^ tissue culture flask (Nunc) at a density of 1 × 10^6^ cells/mL in Iscove's Modified Dulbecco's medium (IMDM) containing 10% heat‐inactivated fetal bovine serum (FBS), penicillin (100 U/mL), streptomycin (100 µg/mL), l‐glutamine (2 mM), recombinant mouse granulocyte‐macrophage colony‐stimulating factor (20 ng/mL), and recombinant mouse IL‐4 (10 ng/mL). Cultures were replaced (50% of the volume) with fresh medium on day 3. Nonadherent cells were collected on day 5 and viable CD11c^+^ BMDCs were enriched with MagniSort mouse CD11c positive selection kit (Invitrogen, Grand Island, NY). Cells were pulsed with HDM (100 µg/mL) in the presence or absence of DMF (75 µM). Overall, 1 × 10^5^ viable CD11c^+^ BMDCs were adoptively transferred in 40 µL of PBS via i.n. administration on day 0 to naïve Balb/c recipient mice. Recipient mice received daily i.n. HDM challenges (50 µg) on days 11 through 13 and end points were analyzed on day 14.

### 2.5 Airway hyper‐responsiveness

Trachea was cannulated with a 19G beveled metal catheter, and airway resistance to increasing concentrations of methacholine (0‐10 mg/mL) was directly measured in mechanically ventilated mice using an Elan RC Fine Pointe system (DSI, St Paul, MN) and mean ± SEM values are presented as cm H_2_O per mL/s.

### 2.6 Analysis of BALF and lung histopathology

Bronchoalveolar lavage (BAL) was performed three times with 0.5 mL of PBS. RBCs were lysed with ACK buffer for 2 minutes at 4°C and cells were resuspended in IMDM medium with 10% FBS. BALF cell counts were performed using a hemocytometer, and differential cell counts were performed on Wright‐Giemsa‐stained cytospin slides using Aerospray Hematology ProSeries 2 instrument (South Logan, UT). In separate experiments, BAL cells were stained for flowcytometry and differential cell count analysis. Lungs were inflated with 10% formalin to pressure of 25 cm H2O, fixed in 10% formalin for 24 hours, dehydrated through gradient ethanol, embedded in paraffin. Lung sagittal sections were cut to thickness of 5 µm and stained with hematoxylin and eosin or periodic acid Schiff (PAS).

### 2.7 HDM‐specific IgE and IgG1

Ninety six‐well plates were coated overnight with 0.01% HDM in PBS and blocked with 1% bovine serum albumin in PBS before the addition of plasma samples that had been diluted 1:5 in blocking buffer and standards for 1 hour. Plates were washed 6× with PBS containing 0.05% Tween‐20 before incubation with biotinylated antimouse IgE or antimouse IgG1 (Pharmingen, San Jose, CA) at a concentration of 2 µg/mL for 1 hour. Next, plates were washed for additional six times, streptavidin‐horseradish peroxidase (R&D Systems) was added for 30 minutes and the amount of bound HDM‐specific antibody was determined using TMB substrate.

### 2.8 Flow cytometry

Lung cells were isolated by enzymatic digestion using type IV collagenase, 1 mg/mL and DNase I, 0.1 mg/mL (Worthington, Lakewood, NJ), in a volume of 2 mL per lung at 37°C for 25 minutes with agitation. Cells were incubated with rat serum to reduce nonspecific binding before surface staining with staining buffer (containing PBS, 3% FBS, 2 mM EDTA, and 10 mM 4‐(2‐hydroxyethyl)‐1‐piperazineethanesulfonic acid) at 4°C for 30 minutes. Lung myeloid cells were identified using antibodies against rat antimouse CD45 efluor 450 (*clone* 30‐F11), CD11c‐APC‐Cy7 (*clone* N418), MHCII‐PE‐Cy7 (*clone* M5/114), SiglecF‐Alexa Fluor 647 (*clone* E50‐2440), CD103‐PerCP‐Cy5.5 (*clone* M290), CD11b‐e‐Fluor 660 (*clone* M1/70), CD64‐PE (*clone* X54‐5/7.1), CD24‐Alexa Fluor 700 (*clone* M1/69), PDCA1‐Alexa Fluor 488 (*clone* e‐Bio 927), all from eBioscience while the fixable viability yellow zombie dye was from BioLegend. CD3^+^ T cells and CD19^+^ B cells present in the mediastinal lymph node (mLNs) and peripheral inguinal lymph node (pLNs) were analyzed using CD3‐Alexa Fluor 647 (*clone* 17‐A2), CD19‐APC‐Cy7 (*clone* eBio1D3). Tregs were analyzed using CD3‐Alexa Fluor 647 (*clone* 17‐A2), CD4‐FITC (*clone* GK1.5), CD8‐e‐Fluor 605 NC (*clone* 53‐6.7) and CD25‐PE‐Cy7 (*clone* PC61.5), all from eBioscience. For quantification of intracellular Foxp3, cells were fixed and permeabilized with Foxp3 staining buffer and reacted with a Foxp3‐PE antibody (*clone* NRRF‐30), both from eBioscience. Broncho alveolar lavage cells were reacted with rat antimouse CD11c‐APC‐Cy7 (*clone* N418), CD3‐Alexa Fluor 647 (*clone* 17‐A2), CD19‐APC‐Cy7 (*clone* eBio1D3), F4/80 (*clone* BM8), all from eBiosciences except antimouse CCR3‐PE (*clone* 83101) (R&D Systems). All antibodies were utilized at a concentration of 0.5‐1 µg/mL. Data were acquired on a CytoFlex‐LX flow cytometer (Beckman Coulter) equipped with 355, 375, 405, 488, 561, 638 and 808 Laser lines using the CytExpert software and analyzed with the Flow Jo software version 10 (Treestar, San Carlos, CA). Cellular debris was excluded using forward light scatter/side scatter plot.

For analysis of intracellular cytokines, single cell suspension of lung cells were suspended in RPMI‐1640 medium supplemented with 10% FBS, l‐glutamine (2 mM), penicillin (100 U/mL) and streptomycin (100 µg/mL), cultured in 24‐well flat bottom plates and stimulated with Cell Stimulation Cocktail (Invitrogen) (containing phorbol 12‐myristate 13‐acetate (PMA), ionomycin, brefeldin A and monensin) for 4 hours at 37°C. Cells were washed with PBS, resuspended in Flow Cytometry Staining Buffer (eBiosciences) containing 10% rat serum (Jackson ImmunoResearch Inc, West Grove, PA) and reacted with 5 µg/mL of rat antimouse CD3‐AF647, and CD4‐FITC (*clone* GK1.5) for 30 minutes, followed by two additional washes. Cells were resuspended in 300 µL of permeabilization buffer (eBiosciences) for 20 minutes. Cells were then reacted with rat antimouse IL‐4‐PE‐Cy7 (*clone* 11B11), IL‐5‐PE (*clone* TRFK5), IL‐13‐Alexa Fluor 488 (*clone* eBio 13A) and IFN‐y‐PerCP‐Cy5.5 (*clone* XMG1.2) (eBiosciences) for 45 minutes at 4°C. Cells were washed twice with permeabilization buffer, resuspended in PBS containing 1% paraformaldehyde and viable CD3^+^/CD4^+^ T cells that expressed IL‐4, IL‐5, IL‐13, and IFN‐γ were enumerated in the CytoFlex‐LX flow cytometer using FMO (fluorescence minus one) as controls using Flow Jo analysis software.

### 2.9 Analysis of DC migration to mLNs

HDM extract (100 µg) was labeled with the Alexa Fluor 647 (AF647) using Protein Labelling Kit (Molecular Probes, Life Technologies) and administered in 50 µL of PBS by i.n. instillation to naïve Balb/c mice 30 minutes after vehicle or DMF treatment (0.5 mg/kg bwt in a total volume of 40 µL). Lungs and mLNs were harvested after 24 hours and the number of Live/SiglecF^−^/CD11c^+^/MHCII ^hi^/SSC^lo^/CD11b^+^/HDM^+^ DCs were quantified with flow cytometry.

### 2.10 Statistics

Data were analyzed using Graph Pad Prism version 7.0a and are presented as mean ± SEM. A one‐way analysis of variance with Bonferroni's or Sidak's multiple comparison test, a Mann–Whitney test or an unpaired *t*‐test were used for analyses. A *P* < .05 was considered significant.

## 3 RESULTS

### 3.1 Lung administration of DMF during HDM challenge phase abrogates the cardinal features of allergic asthma

**Figure 1:**
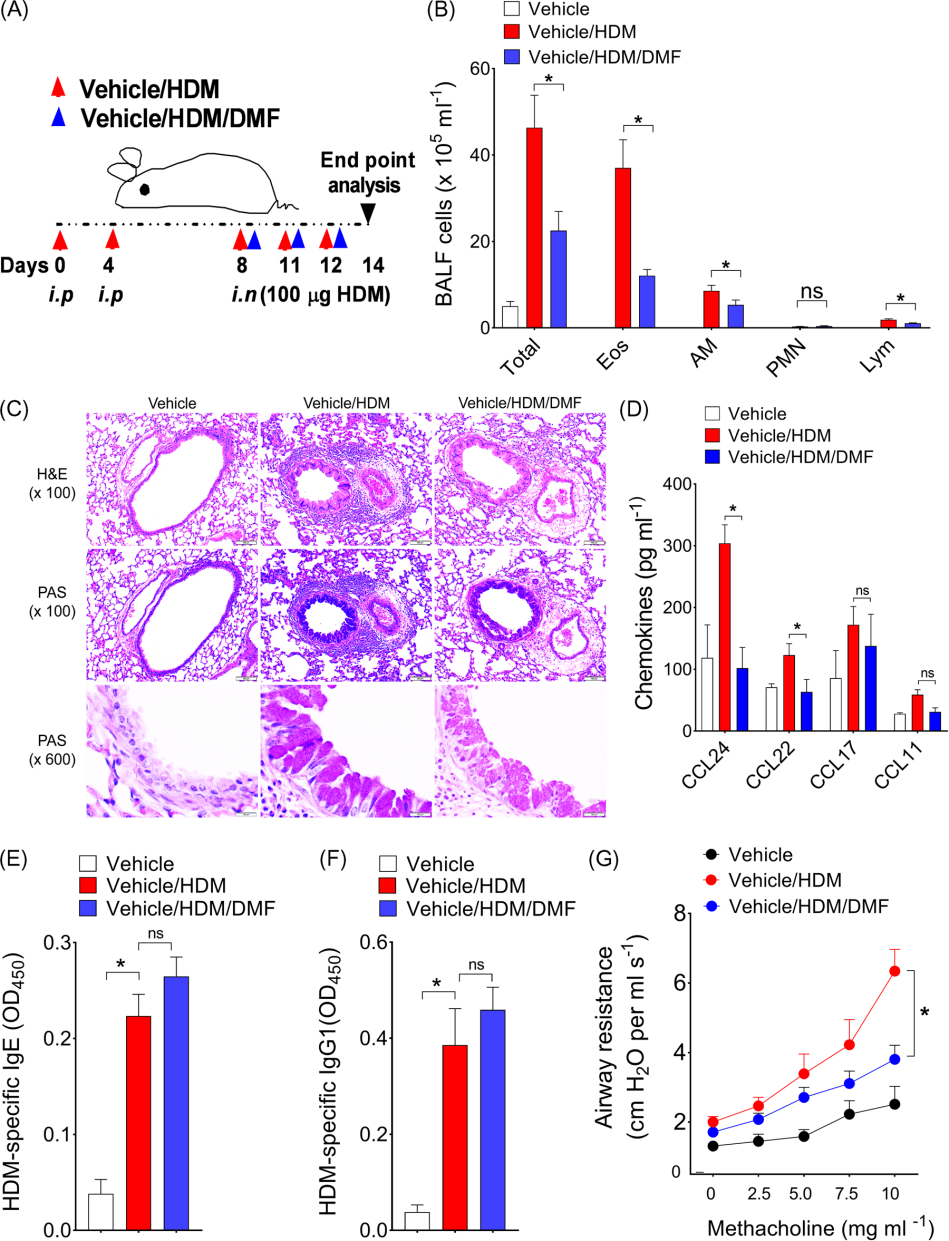
Effect of local DMF treatment on asthma features. A, Mice were sensitized with two i.p. injections of 100 μg HDM on days 0 and 4 and challenged on days 8, 11, and 12 by intranasal administration of HDM (100 μg) before harvest and endpoint analysis on day 14. Thirty minutes before each HDM challenge, mice received an i.n. administration of vehicle or DMF (dosage at 0.5 mg/kg bwt) in volume of 40 µL). B, The number of total BALF inflammatory cells and inflammatory cell types (Eos, AM, PMN, and Lym from vehicle, HDM, or DMF treated mice (*n* = 8‐10 mice, *P < .01, vehicle/HDM‐challenged versus Vehicle/HDM/DMF, one‐way ANOVA with Sidak's multiple comparison test). C, Representative lung histology sections stained with H&E or PAS are shown. Scale bars = 100 μm for the ×100 and ×600 images. D, BALF levels of CC‐chemokine ligands from mice treated or not with DMF (*n* = 5‐10 mice, *P < .05, or *P* = NS, unpaired *t*‐test). Results shown are pooled data from two independent experiments. E,F, Plasma levels of HDM‐specific IgE and IgG1 (*n* = 9‐10 mice, *P < .01, Mann–Whitney test, vehicle/HDM‐challenged versus DMF‐treated mice). G, Airway resistance (cm H_2_O per mL/s) to increasing dosage of inhaled methacholine from vehicle, HDM‐challenged or DMF treated mice (*n* = 8‐10 mice) (*P < .05, two‐way ANOVA). AM, alveolar macrophages; DMF, dimethyl fumarate; Eos, eosinophils; HDM, house dust mite; H&E, hematoxylin and eosin; Lym, lymphocytes; PAS, periodic acid‐Schiff; PMN, neutrophil

First, we considered whether local administration of DMF before HDM challenge would impact the manifestations of allergic asthma in already sensitized mice. In these experiments, mice were sensitized with HDM, followed by multiple i.n. HDM challenges with or without local DMF administration (Figure 1A). Bronchoalveolar lavage fluid (BALF) cells and their differential analysis were enumerated. As shown in Figure 1B, the number of BALF inflammatory cells recovered from HDM‐challenged mice that received DMF treatment were significantly reduced as compared with those from vehicle treated mice, which represented decreases in eosinophils (Eos), alveolar macrophages (AM), and lymphocytes (Lym). Similarly, treatment with DMF before each HDM challenge showed a decrease in the extent of peribronchial and perivascular inflammatory cell infiltrates and mucous cell metaplasia in lung sections compared with HDM‐challenged control (Figure 1). BALF levels of C‐C chemokine ligands, CCL24 and CCL22 were also significantly reduced with DMF treatment as compared with untreated control, whereas there was no difference in levels of CCL17 and CCL11 (Figure 1D). HDM sensitized mice treated with either DMF or vehicle showed no change in plasma levels of HDM‐specific IgE (Figure 1E) or IgG1 (Figure 1F). In addition to the reduction in airway inflammation, DMF treatment also significantly attenuated HDM‐mediated increases in airway hyperresponsiveness over the complete dose range with inhaled methacholine (AHR; Figure 1G). The HDM‐challenged mice showed higher average of airway resistance with increasing methacholine dosage compared with vehicle only, but this airway hyperresponsiveness was significantly decreased by local DMF treatment. Collectively, these findings demonstrate that local administration of DMF during the challenge phase can attenuate airway inflammation and cardinal manifestations of experimental HDM‐induced asthma, including mucous metaplasia and AHR.

### 3.2 DMF treatment alters lung myeloid cell distribution and attenuates Th2 inflammation

**Figure 2:**
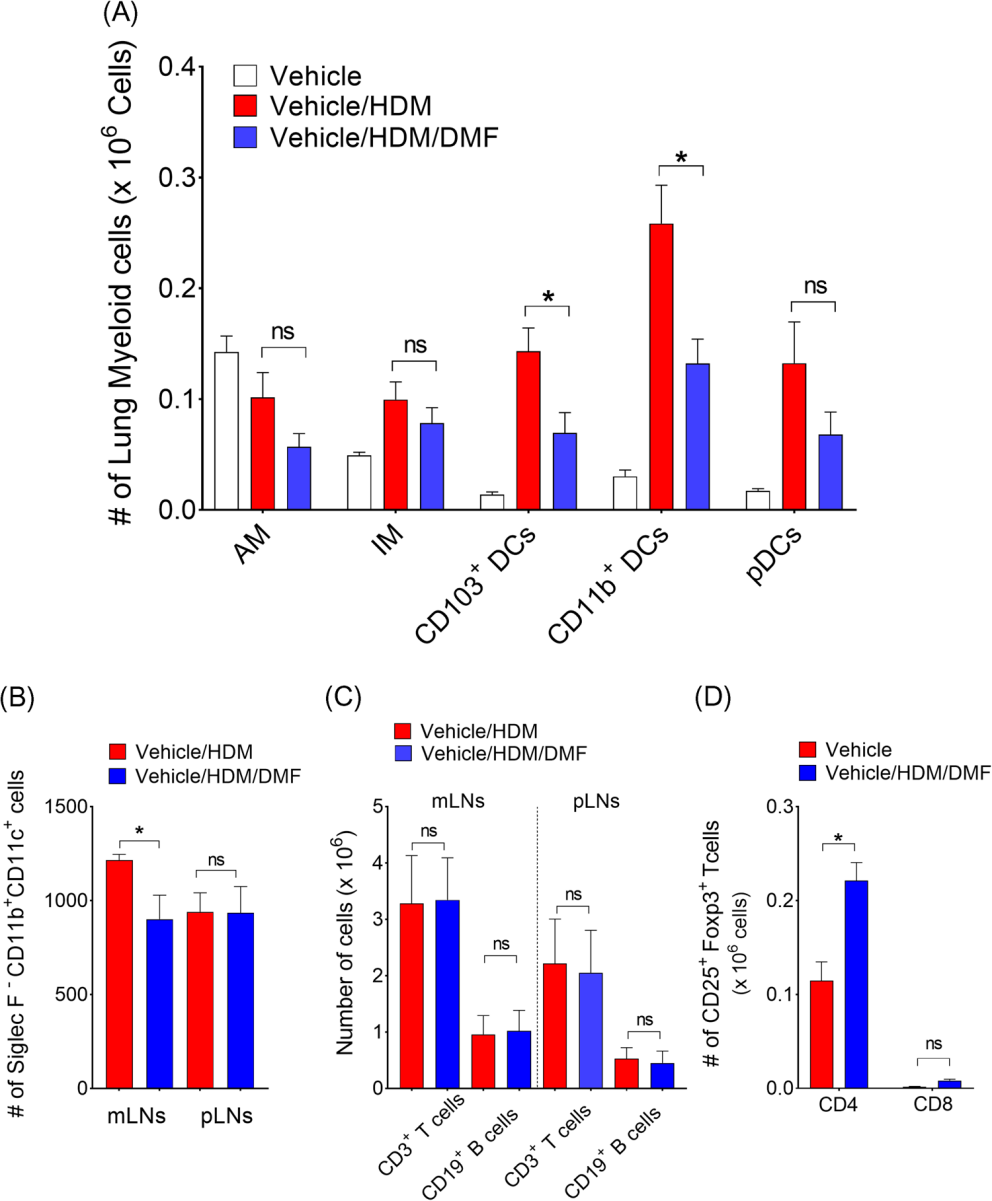
Effect of local DMF treatment on lung myeloid cell distribution. A, Changes of myeloid‐cell subsets during HDM‐induced airway inflammation and DMF treatment. Single cell suspension of enzymatically digested mouse lungs were prepared and myeloid‐cell subsets were identified and enumerated. Difference between groups were compared using one‐way ANOVA with Sidak's multiple comparison test (*P < .01 or *P* = NS, vehicle/HDM‐challenged versus vehicle/HDM/DMF). B, Enumeration of CD11b^+^ DCs, (C) T and B cells from lung draining mLNs or pLNs, and (D) CD25^+^/FoxP3^+^ Tregs (CD4^+^ and CD8^+^) in draining mLNs from HDM‐challenged and DMF treated mice*.* *P < .05 or *P* = NS, vehicle/HDM‐challenged versus vehicle/HDM/DMF, one‐way ANOVA with Sidak's multiple comparison test). Data are representatives of at least two independent experiments and represents means ± SEMs (*n* = 8‐10 mice). DMF, dimethyl fumarate; HDM, house dust mite; mLV, mediastinal lymph node; pLN, peripheral lymph node

Since, local treatment with DMF was effective in suppressing airway inflammation and asthma features, experiments were next conducted to assess the lung myeloid cell distribution in response to HDM challenge that might be directly responsible for the treatment effect. First, we assessed whether the myeloid cell subsets, B and T cells were altered with DMF treatment in lungs, draining mediastinal lymph nodes (mLNs) and peripheral lymph nodes (pLNs) compared to vehicle control. To delineate different lung myeloid cell subsets, we used a modified gating strategy.^27^ As shown in Figure 2A, interstitial macrophages (IM), CD11b^+^ conventional DCs (cDC2), CD103^+^ conventional DCs (cDC1) and plasmacytoid DCs numbers in the lungs were induced with HDM allergen challenge as compared with vehicle treated naïve controls. Lung recruitment of CD11b^+^ cDC2, CD103^+^ cDC1, and interstitial macrophages were markedly decreased with DMF treatment compared to untreated control mice. Second, we found a reduction of a CD11b^+^ cDC2 numbers in lung draining mLNs, whereas the number of CD11b^+^ cDC2 in pLNs was not altered with local DMF treatment (Figure 2B). There was no change in the T or B cell numbers from mLNs and pLNs with DMF treatment compared to untreated control (Figures 2C and S3A). We also assessed whether the number of CD3^+^/CD4^+^/CD25^+^/Foxp3^+^ regulatory T cells (Tregs) was modified with DMF treatment. However, we found that there was an increase of CD4^+^ Tregs number from mLNs of HDM‐challenged DMF treated mice as compared to untreated (Figure 2D).

**Figure 3:**
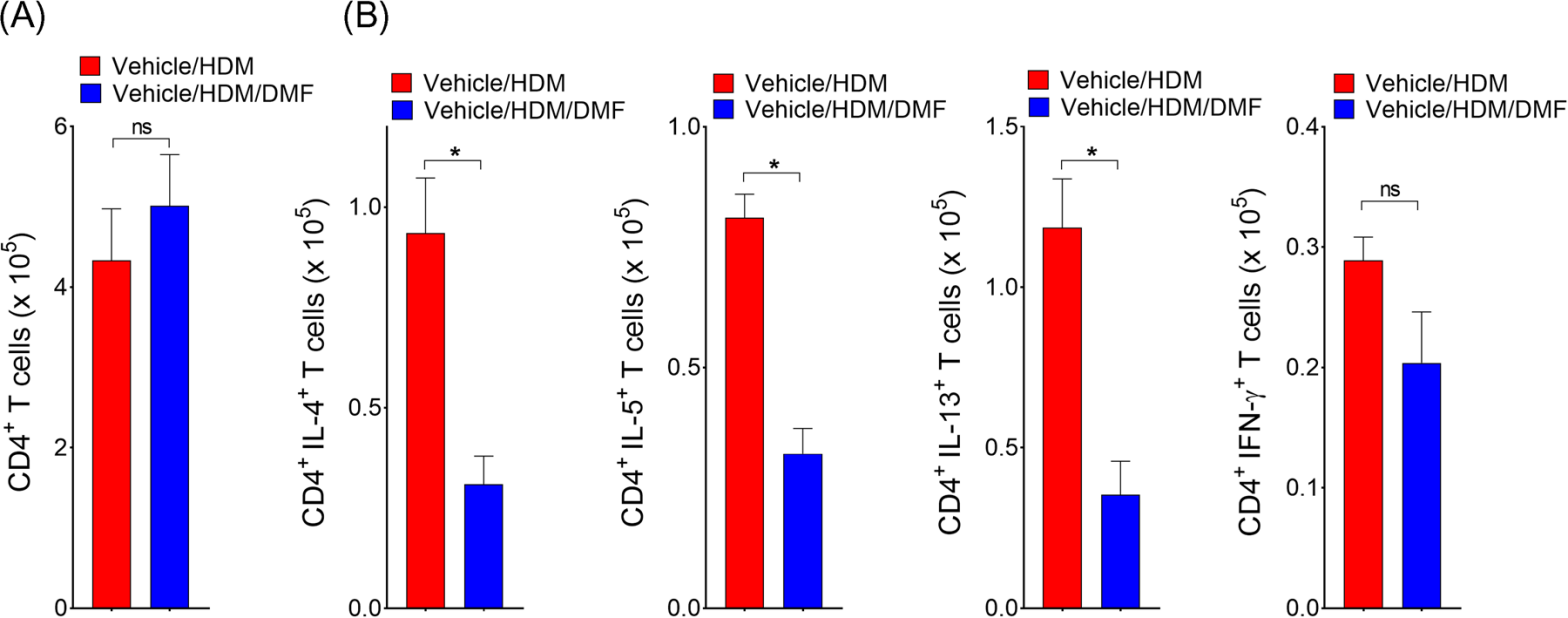
Local DMF administration attenuates CD4^**+**^ Th2‐cytokine producing cells in lungs. A, The total number of CD4^+^ T cells, as well as (B) IL‐4^+^/CD4^+^, IL‐5^+^/CD4^+^, IL‐13^+^/CD4^+^, and IFN‐γ^+^/CD4^+^ T cells in lung, was quantified by flowcytometry (*n* = 9‐10 mice, *P < .01 or *P* = NS, Mann–Whitney test, vehicle/HDM‐challenged versus DMF‐treated mice). Results are pooled data from at least two independent experiments. DMF, dimethyl fumarate; HDM, house dust mite

Additional experiments were performed to characterize further the effects on Th1 and Th2‐cytokine producing lung cells. Although, there were no changes in lung CD4^+^ T cell numbers (Figure 3A), local DMF treatment strikingly reduced IL4^+^, IL5^+^, and IL13^+^ Th2‐cytokine producing effector cells in the lungs as compared to HDM challenged vehicle control (Figure 3B). There were no difference in IFN‐γ^+^ Th1‐cytokine producing cell numbers. Collectively, these findings demonstrate that local administration of DMF during the challenge phase can attenuate the recruitment of lung myeloid DCs, thereby suppress Th2 mediated airway inflammation in response to inhaled HDM.

### 3.3 DMF treatment inhibits migration of lung CD11b^+^ cDC2 to draining mediastinal lymph node

**Figure 4:**
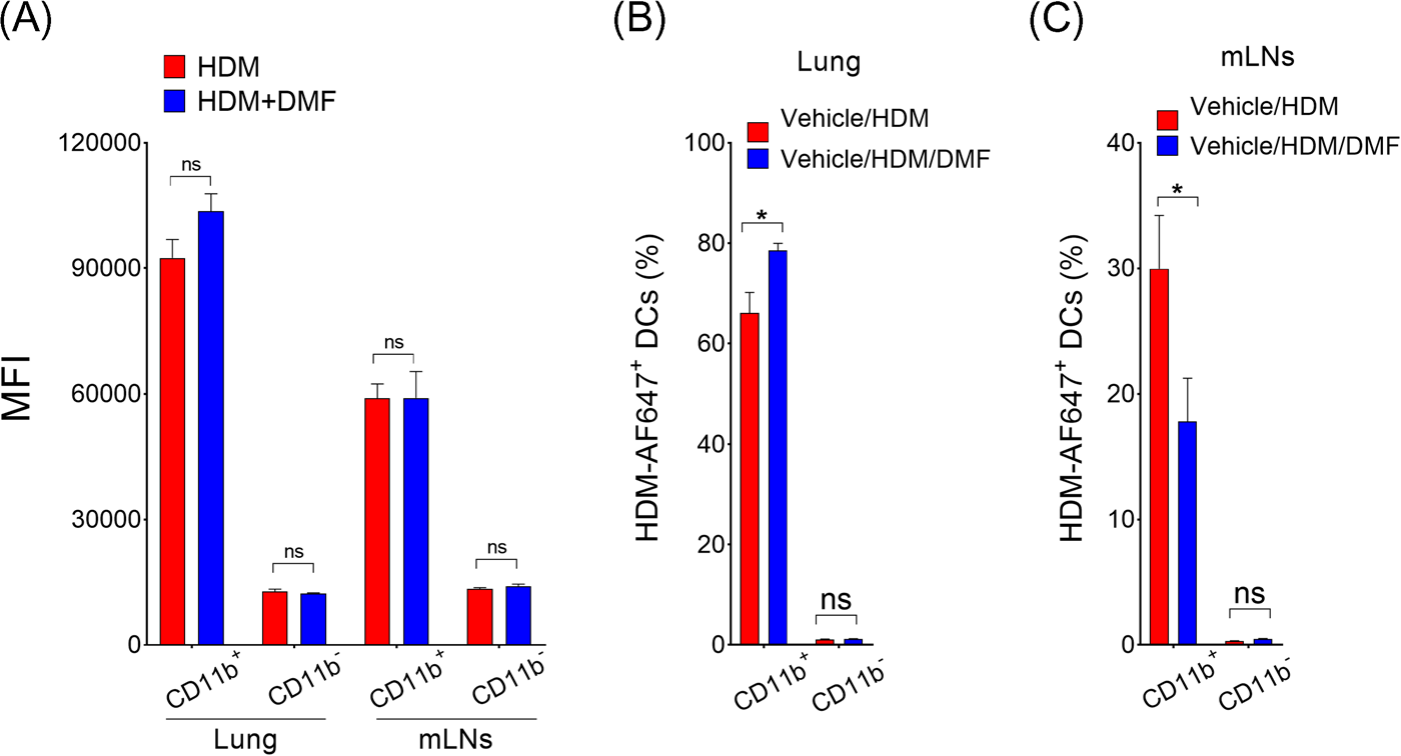
Effect of local DMF administration on lung DC migration to the lung draining mLNs. On day 0, mice were administered with HDM extract (100 μg) labeled with Alexa Fluor 647 with or without DMF (10 μg) treatment. A, MFI and (B,C) percent of HDM‐AF647^+^ CD11b^+^ and CD11b^−^ DCs from lung and draining mLNs were enumerated 24 hours after installation of labeled HDM. SiglecF^−^/CD11c^+^/MHCII^hi^/AF647^+^ DCs were gated for evaluation (*n* = 8‐12 mice per group, *P < .01, or *P* = NS, unpaired *t*‐test, vehicle/HDM‐challenged versus DMF‐treated mice). Data represents means ± SEMs. DC, dendritic cell; DMF, dimethyl fumarate; HDM, house dust mite

Treatment with DMF reduced the total numbers of CD11b^+^ cDC2 in the draining mLNs, which is an indicative of the in vivo effect on the migratory capacity of lung DCs. Next, to assess the functional significance of the reduction in numbers of DCs, AF647‐labeled HDM was administered to the lungs thirty minutes after vehicle or DMF treatment. Twenty‐four hours following instillation the number of HDM‐AF647^+^ DCs were enumerated in the lung or in draining mLNs. The individual cell intensity of HDM‐AF647 staining showed no change in lung or mLNs with vehicle or DMF treatment, indicating that DMF administration did not interfere with the HDM uptake process (Figure 4A). DMF treatment significantly increased the retention of HDM‐AF647^+^ CD11b^+^ cDC2 in the lung epithelium (Figure 4B). In addition, exposure to DMF inhibited the migration of lung CD11b^+^ cDC2 to the draining mLNs (Figure 4C). Altogether, these experiments show that local DMF treatment interfere with the migration process of DCs from lung epithelium to the draining mLNs, which is an integral step to generate the immune response and Th2 effector function in response to inhaled HDM.

### 3.4 DCs‐driven airway inflammation is suppressed by DMF treatment

Having shown that DMF significantly influence DCs in HDM‐challenged phase, experiments were next conducted to assess the effect of DMF on DC‐related inflammatory genes and transcription factors (Figure S1A). HDM stimulation induced an increase in IL10, ICOS‐L and ST2 gene expression in DCs as compared to vehicle control, whereas the reduction in expression of these genes were not statistically significant with DMF treatment. DMF significantly suppresses HDM‐induced OX40‐L expression in DCs that elicits costimulatory signal and development of Th2 responses.^28^ We also show that the transcription factor basic leucine zipper transcription factor ATF‐like 3 (Batf3), which plays nonredundant function in DC development were significantly reduced with DMF treatment (Figure 1B).

**Figure 5:**
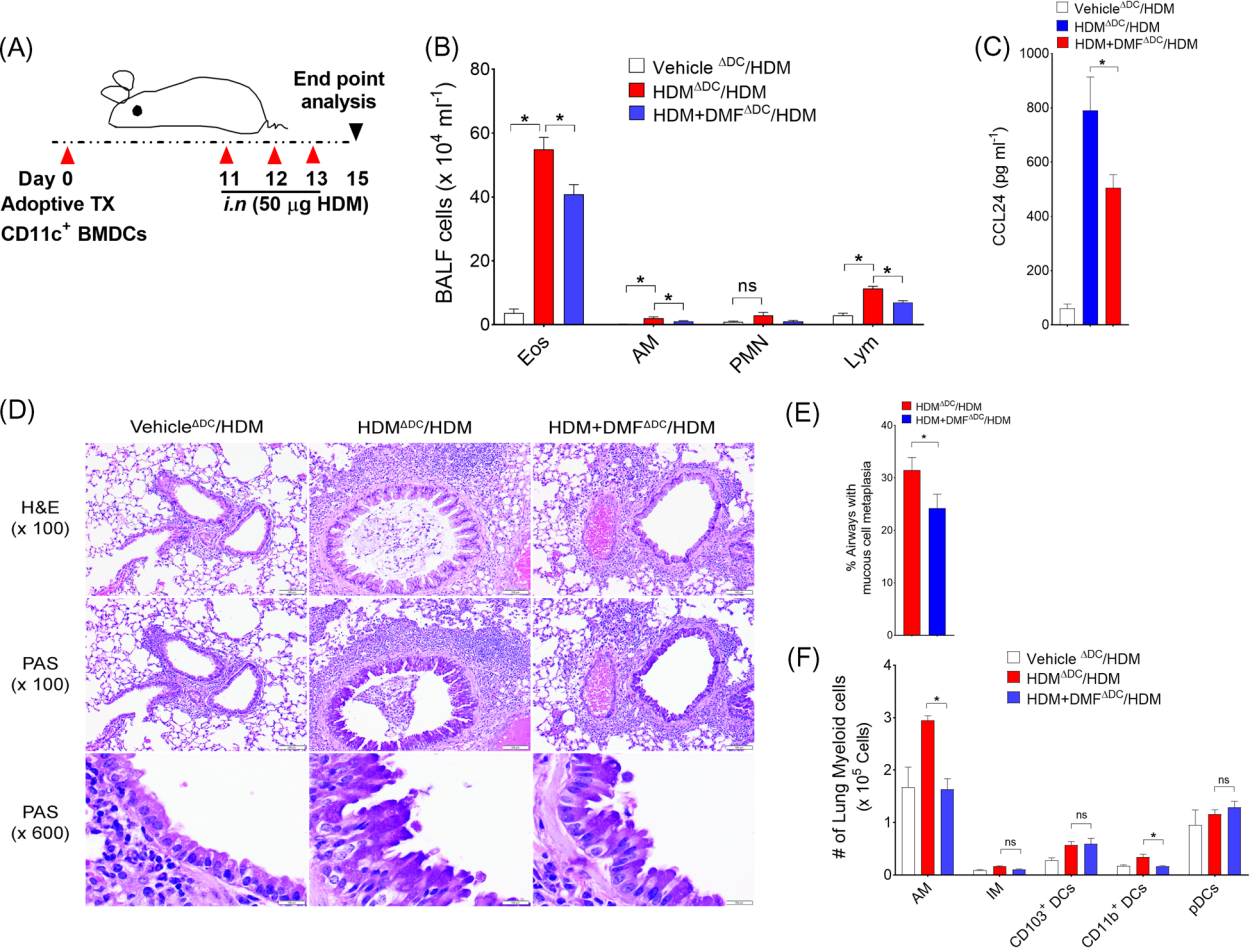
The adoptive transfer of HDM‐pulsed CD11c^+^ BMDCs treated with DMF have an impaired ability to induce allergen‐mediated airway inflammation. A, DMF treated BMDCs were pulsed ex vivo with vehicle or HDM (100 µg/mL) for 16 hours. CD11c^+^ DCs (0.1 × 10^6^) were adoptively transferred to naïve Balb/c recipient mice by means of intranasal administration on day 0, and intranasal HDM challenges (50 µg) were administered on day 11 through day 13, to all recipient mice before endpoint analysis on day 15. B, Enumeration of BALF inflammatory cell subtypes in recipient mice by flow cytometry. BALF lymphocytes (*lym*) were identified as CD3^+^/CD45R^+^/MHCII^−^ cells, and the CD3^−^/CD45R^−^ cell population were gated as CD11c^+^/MHCII^+^/F4/80^+^ alveolar macrophages (*AM*); F4/80^−^/SSC ^hi^/CCR3^−^ neutrophil (*PMN*); and SSC ^hi^/MHC II^−^/CCR3^+^ eosinophil (*Eos*) (*n* = 8‐16 mice per group). *P < .05, or *P* = NS, HDM‐pulsed DMF treated versus HDM‐pulsed, or HDM‐pulsed versus vehicle, one‐way ANOVA with Sidak's multiple comparison test*.* C, BALF levels of CCL24 from recipient mice that received HDM‐pulsed DMF‐treated DCs or HDM‐pulsed untreated DCs. *P < .05, HDM‐pulsed DMF treated versus HDM‐pulsed, unpaired *t*‐test, (*n* = 4‐8 mice per group). D, Representative histologic lung sections stained with H&E and PAS. Scale bars = 100 μm for the ×100 and ×600 images. E, Quantitation of PAS^+^ mucous cell metaplasia. F, Lung myeloid cells in recipient mice sensitized with HDM‐pulsed DMF‐treated DCs or HDM‐pulsed untreated DCs. Results are representative of least two independent experiments and expressed as means ± SEMs (*n* = 4). Difference between groups were compared using one‐way ANOVA with Sidak's multiple comparison test (*P < .01 or *P* = NS, HDM‐pulsed DMF treated versus HDM‐pulsed). DC, dendritic cell; DMF, dimethyl fumarate; H&E, hematoxylin and eosin; HDM, house dust mite

**Figure 6:**
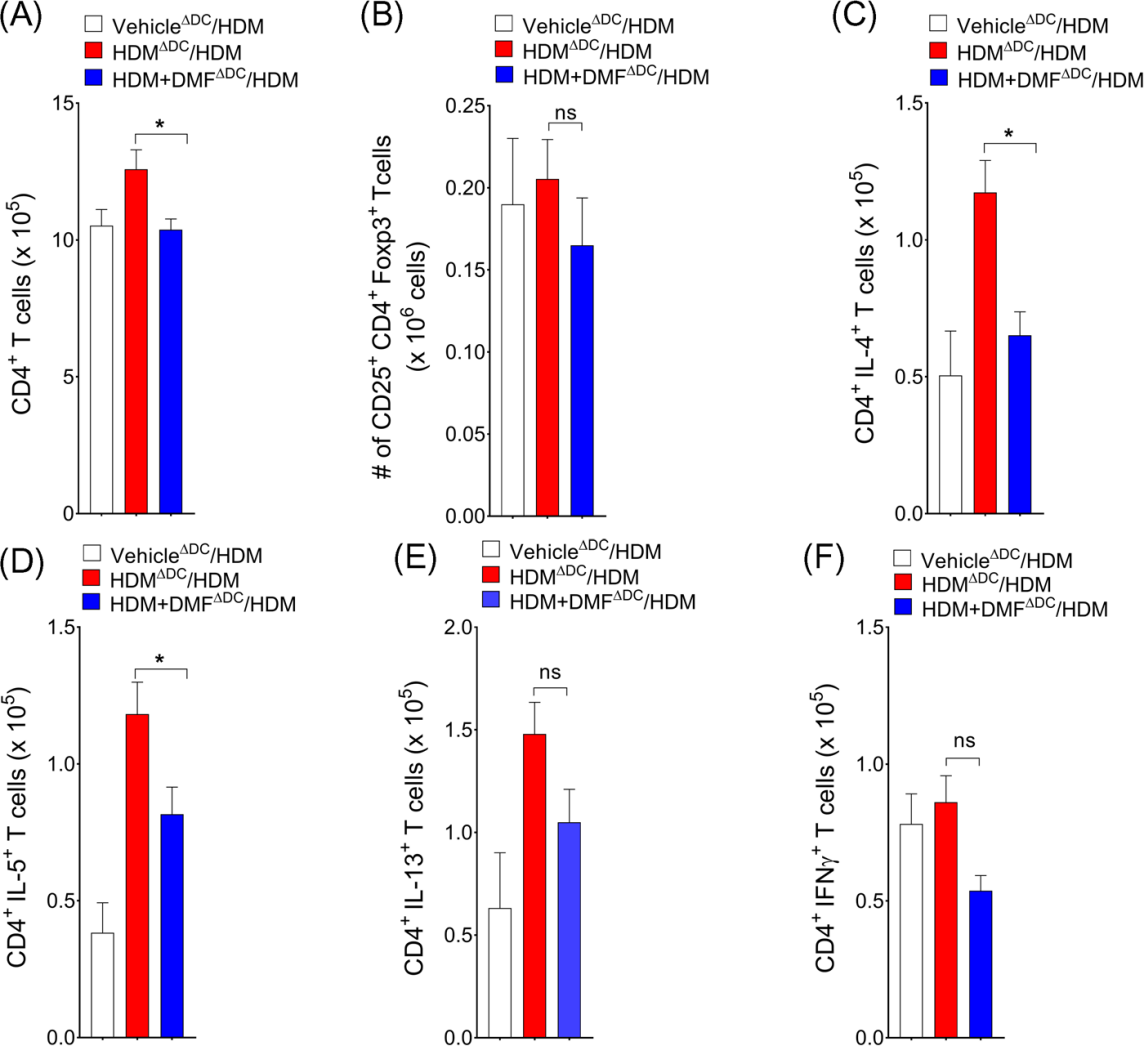
DMF treated DCs induces Th2 immune responses. The total number of (A) lung CD4^+^ T cells and (B) CD4^+^/CD25^+^/FoxP3^+^ Tregs in mediastinal LNs, as well as (C) IL‐4^+^/CD4^+^, (D) IL‐5^+^/CD4^+^, (E) IL‐13^+^/CD4^+^, and (F) IFN‐γ^+^/CD4^+^ T cells from recipient mice lungs that received HDM‐pulsed DMF‐treated or untreated DCs (*n* = 4‐8 mice per group). Results are representative of at least two independent experiments. One‐way ANOVA with Sidak's multiple comparison test (*P < .01 or *P* = NS, HDM‐pulsed DMF treated versus HDM‐pulsed). DC, dendritic cell; DMF, dimethyl fumarate; HDM, house dust mite; Th2, T helper 2

Next, adoptive transfer experiments were performed to confirm that the attenuated HDM‐induced airway inflammatory responses with local DMF administration were mediated specifically by DCs. CD11c^+^ bone marrow derived dendritic cells (BMDCs) pulsed with vehicle or DMF and then transferred adoptively to naïve mice that subsequently received multiple i.n. HDM challenges to induce DC‐driven allergic airway inflammation (Figure 5A). As a marker for inflammation in the lungs, BALF cells and their differential analysis were enumerated using multicolor flow cytometry and sequential gating analysis (Figure S3B). Lymphocytes (*Lym*) were identified as CD3^+^/CD45R^+^/MHCII^−^ cells, and the CD3^**−**^ **/**CD45R^**−**^ cell population were gated as CD11c^+^/MHCII^+^/F4/80^+^ alveolar macrophages (*AM*); F4/80^−^/SSC^hi^/CCR3^−^ neutrophil (*PMN*); and SSC^hi^/MHCII^−^/CCR3^+^ eosinophil (*Eos*). Recipients of adoptively transferred HDM‐pulsed DCs that had been treated with DMF had significant reductions in the number of BALF inflammatory cells as compared with recipients of HDM‐pulsed DCs that had been treated with vehicle alone (Figure 5B). BALF levels of CCL24, as a proxy measure of eosinophil infiltration, were significantly reduced in recipients of DMF‐treated HDM‐pulsed DCs compared to vehicle (Figure 5C). Consistent with this, lung histology showed a reduction in peribronchial inflammatory cell infiltrates in recipient of HDM pulsed DCs that had been treated with DMF, which was associated with a decrease in mucous cell metaplasia (Figure 5D). Mice that received the adoptive transfer of HDM pulsed CD11c^+^ BMDCs and had been treated ex vivo with DMF demonstrated significant reductions in the lung alveolar macrophages and CD11b^+^ cDC2 (Figure 5E). Although there was reduction of CD80 and dectin‐1 expressions in lung DCs from recipients that adoptively received DMF‐treated DCs, these differences appeared modest in the cell surface expression level of CD86 and dectin‐2 (Figure S2). Lastly, recipients of DMF treated HDM pulsed DCs had reductions in number of CD4^+^‐IL4^+^ and ‐IL5^+^ Th2 cytokine producing lung cells, as compared with recipients of HDM‐pulsed DCs that had not been pulsed with DMF (Figure 6). Collectively, these results demonstrate that DMF‐mediated pharmacological modulation of DCs impairs their ability to initiate allergic sensitization and Th2‐driven airway inflammatory responses.

## 4 DISCUSSION

Airway DCs are primary antigen presenting cells that initiate and maintain allergic sensitization and Th2 adaptive immune responses to inhaled aeroallergen in asthma.^24^ DCs in the lung are primarily comprises of two main subsets of conventional DCs (cDCs) and tolerogenic plasmacytoid DCs (pDCs). Type 1 cDCs that express α integrin CD103^+^ (cDC1) primarily cross present antigen to naïve CD8^+^ T cells, inferior in their antigen uptake capacity, and are redundant for Th2 immune response.^29^, ^30^ In contrast, type 2 conventional DCs (cDC2) are CD11b^+^ and efficiently take up and process aeroallergen, such as HDM, and migrate to the draining mediastinal lymph nodes to induce T cell differentiation and production of effector cytokines.^31^, ^32^ Consistent with this, mice depleted of DCs during HDM challenge phase were unable to mount Th2 responses to HDM, whereas adoptive transfer of DCs were sufficient to induce Th2 immunity and restore airway features.^24^, ^26^ Therefore, airway DCs are attractive therapeutic targets for development of antiasthmatic drugs.

Limited or conflicting data, however, exist regarding the mechanism of DMF action and their effect on DC function.^33^, ^34^ Prior studies using monocyte derived DCs have found that DMF inhibited the GMCSF and IL4 driven maturation and differentiation process by inducing apoptosis, thereby failed to proliferate lymphocytes.^34^, ^35^ A recent study, however, have shown that DMF increases HO expression and deplete the intracellular pool of reduced glutathione (GSH) level, and promotes IL10 production to induces IL4^+^ autoreactive Th2 cells in Th1 and Th17‐mediated autoimmune diseases.^16^ This immune deviation is mediated by inhibition of STAT1 phosphorylation and suppression of IL12 and IL23 production. However, the inhibition of Th1/Th17 cells and induction of IL10 producing DCs and IL4^+^Th2 cells appears after more than 4 weeks of DMF therapy for clinical improvement.^36^, ^37^ In contrast, short‐term feeding of DMF to rodents for 2 weeks was shown to increase GSH level and inhibits phosphorylation and ubiquitination of IκB and induces NF‐κB activation.^38^, ^39^ However, the immunomodulatory effects of DMF on DC‐mediated allergic airway inflammation and adaptive Th2 immunity has not been elucidated previously.

Here we investigated whether local administrations of DMF is effective to suppress DC‐mediated induction and allergic sensitization of airway inflammation and Th2‐mediated adaptive immune response. First, we show that i.n. administration of DMF in HDM challenge phase attenuate airway inflammation, mucous cell metaplasia, airway resistance to inhaled methacholine. Furthermore, DMF administration increased recruitment of lung draining mLNs Tregs. This is consistent with prior reports demonstrating local administration of the oral immunosuppressant small molecule sphingosine 1‐phosphate agonist FTY720 via inhalation are effective to attenuate allergic airway inflammation and Th2 immune response without accompanying lymphopenia as serious adverse effect.^40^ However, we were unable to found any significant changes in the lung Treg population with DMF treatment. In particular, FTY720 inhibit the migration of airway DCs from epithelium to the draining mediastinal lymph nodes, thereby suppress Th2 immune response in a murine model of experimental asthma.^40^, ^41^ We also show that the immunomodulatory small molecule DMF attenuates all manifestations of HDM‐induced allergic asthma when administered before or during HDM challenge. This is also highly relevant to the DMF effect, which suppressed airway inflammation without altering the lymphocyte distribution in the lung and in the periphery. We also showed that local DMF application significantly inhibited the lung recruitment of CD11b^+^ cDC2 and suppress cytokine secreting CD4^+^IL4^+^, CD4^+^ IL5^+^, and CD4^+^IL13^+^ Th2 effector cells. Therefore, we propose that in presence of DMF, DCs might have an impaired ability to induce Th2 mediated adaptive immune responses to HDM.

Here we demonstrate that the number of mediastinal LNs DCs are significantly reduced with inhaled DMF treatment, which could be resultant of reduced airway inflammation in the HDM challenged lung and associated reduced influx of lung DCs into the draining mLNs.^42^ We demonstrate that single DMF treatment before administration of fluorescent labeled HDM to naïve mice inhibit the migration of CD11b^+^ cDC2 to the draining mLNs associated with an accumulation of DCs in the lung tissues. In line with others, DMF and its active MMF interfere with adhesion molecule expression and inhibit leukocyte chemotaxis in a hydroxycarboxylic acid receptor 2 (HCA_2_)‐dependent manner, thereby exerts its protective effect.^43^, ^44^, ^45^

We also assessed whether pharmacological treatment of DCs with DMF are effective to ameliorate DCs‐driven allergic sensitization and airway inflammation. First, we showed that the adoptive transfer of HDM‐pulsed CD11c^+^ BMDCs treated with DMF had an impaired ability to induce allergic sensitization, as indicated by reduced BALF inflammatory cell infiltration associated with reductions in CCL24 level, as well as reduced airway inflammation and mucous cell metaplasia. CD11b^+^ cDC2 in the lung are known for their superior antigen presenting capacity and induce Th2 responses by expression of IRF4 (interferon regulatory factor‐4) target genes.^31^, ^46^ Exposure to HDM is recognized by the innate C‐type lectin receptors dectin 1 and dectin 2, which are present on cDC2 and induces chemokine receptor expression and migration of DCs to mLNs.^47^, ^48^, ^49^ Consistent with this, in response to HDM challenge dectin1^−/−^ mice failed to mount Th2 immune response and eosinophilic airway inflammation, and showed reduced CCR7 expression on cDC2 and less migration of DCs to mLNs.^49^ Similarly, mice that received DMF treated DCs displayed a phenotype of reduced cell surface expression of the costimulatory molecule CD80 and dectin 1, as well as reduced numbers of CD4^+^ Th2 cytokine producing cells, with no effect on CD4^+^ IFN‐γ^+^ T cells. Furthermore, we found that DMF treatment significantly reduced the HDM‐induced TNF‐superfamily member OX40‐L expression in DCs, which are required for Th2 cell differentiation.^50^

In summary, we have identified a novel mechanism of DMF action in the lung, where it effectively interferes with DC migration to the draining mLNs and subsequent induction of allergic sensitization and Th2 immunity to HDM. Our results in this report validates the concept, for what we believe for the first time, that lung DMF administration inhibits the Th2‐driven cardinal manifestation of allergic asthma by altering the airway DCs function without causing systemic lymphopenia. This identifies the mechanistic rationale of the small molecule drug DMF to target airway DCs in allergic asthma.

## CONFLICT OF INTERESTS

The authors declare that there are no conflict of interests.

## AUTHOR CONTRIBUTIONS

AKJ and AM designed research together with AKJ, AM, and MS, performed the experiments. AJ, AM, and MS, analyzed the data. AM, AKJ, AS, and RCC contributed to the writing and reviewing the final manuscript.

## DATA ACCESSIBILITY

All the data presented here are new and fully accessible.

## ETHICS STATEMENT

Murine experimental protocols were approved by the Animal Care and Use Committee of the Auburn University (Auburn, AL).

## Supporting information


***Supplementary Figure 1.*** **Effect of DMF treatment on costimulatory and transcription factor gene expressions in DCs.** Splenic DCs were pulsed with HDM (100 µg ml^−1^) and were cultured overnight in presence or absence of DMF (75 µM). DCs‐specific relative expression of **A)** HDM‐driven immune pathway and **B)** transcription factor genes are shown. Results are pooled from at least 2 independent experiments and values represent means ± SEMs. (n = 4‐6, *P < 0.05, or *P* = ns, unpaired t‐test, HDM versus HDM+DMF‐treated DCs)

***Supplementary Figure 2.*** **Activation profile of lung DCs from recipient mice sensitized with DMF treated BMDCs differs from untreated mice.** Cell surface expression of mean fluorescence intensity (MFI) of **A)** CD80 **B)** CD86 **C)** Dectin‐1, and **D)** Dectin‐2 by lung SiglecF /SSC^lo^ / CD11c^+^ / MHCII ^hi^ DCs from recipient mice that received HDM‐pulsed BMDCs either DMF‐treated or untreated and challenged with HDM. Results are representative of data generated in two different experiments (n= 4 mice /group)

***Supplementary Figure 3.*** **Gating strategy for enumeration of immune cells in lymph nodes and BALF sample.** CD11b^+^ DC were identified in mLNs and pLNs samples as SiglecF^‐^/CD11c^+^/CD11b^+^ **(A)**, and **(B)** differential counting of BALF cells were analyzed by flow cytometry using sequential gating analysis. Lymphocytes (*Lym*) were identified as CD3^+^/ CD45R^+^/ MHCII^‐^ cells, and the CD3^**‐**^ **/** CD45R^**‐**^ cell population were gated as CD11c^+^/ MHCII^+^/ F4/80^+^ alveolar macrophages (*AM*); F4/80^‐^/ SSC ^hi^/ CCR3^‐^ neutrophil (*PMN*); and SSC ^hi^/ MHC II^‐^/ CCR3^+^ eosinophil (*Eos*)

## References

[1] Lehmann JC , Listopad JJ , Rentzsch CU , et al. Dimethylfumarate induces immunosuppression via glutathione depletion and subsequent induction of heme oxygenase 1. J Invest Dermatol. 2007;127:835‐845. 17235328 10.1038/sj.jid.5700686

[2] Michell‐Robinson MA , Moore CS , Healy LM , et al. Effects of fumarates on circulating and CNS myeloid cells in multiple sclerosis. Ann Clin Transl Neurol. 2016;3:27‐41. 26783548 10.1002/acn3.270 PMC4704479

[3] Linker RA , Haghikia A . Dimethyl fumarate in multiple sclerosis: latest developments, evidence and place in therapy. Ther Adv Chronic Dis. 2016;7:198‐207. 27433310 10.1177/2040622316653307 PMC4935836

[4] Gold R , Kappos L , Arnold DL . Placebo‐controlled phase 3 study of oral BG‐12 for relapsing multiple sclerosis. New Engl J Med. 2012;367:1098. 22992073 10.1056/NEJMoa1114287

[5] Havrdova E , Giovannoni G , Gold R , et al. Effect of delayed‐release dimethyl fumarate on no evidence of disease activity in relapsing‐remitting multiple sclerosis: integrated analysis of the phase III DEFINE and CONFIRM studies. Eur J Neurol. 2017;24:726‐733. 28328179 10.1111/ene.13272 PMC5413827

[6] Kappos L , Gold R , Miller DH , et al. Investigators BGPIS: Efficacy and safety of oral fumarate in patients with relapsing‐remitting multiple sclerosis: a multicentre, randomised, double‐blind, placebo‐controlled phase IIb study. Lancet. 2008;372:1463‐1472. 18970976 10.1016/S0140-6736(08)61619-0

[7] Kornberg MD , Bhargava P , Kim PM , et al. Dimethyl fumarate targets GAPDH and aerobic glycolysis to modulate immunity. Science. 2018;360:449‐453. 29599194 10.1126/science.aan4665 PMC5924419

[8] Blewett MM , Xie JJ , Zaro BW , et al. Chemical proteomic map of dimethyl fumarate‐sensitive cysteines in primary human T cells. Sci Signal. 2016;9:rs10. 27625306 10.1126/scisignal.aaf7694 PMC5068918

[9] Blatnik M , Frizzell N , Thorpe SR , Baynes JW . Inactivation of glyceraldehyde‐3‐phosphate dehydrogenase by fumarate in diabetes—Formation of S‐(2‐succinyl)cysteine, a novel chemical modification of protein and possible biomarker of mitochondrial stress. Diabetes. 2008;57:41‐49. 17934141 10.2337/db07-0838 PMC2423377

[10] Schulze‐Topphoff U , Varrin‐Doyer M , Pekarek K , et al. Dimethyl fumarate treatment induces adaptive and innate immune modulation independent of Nrf2. Proc Natl Acad Sci U S A. 2016;113:4777‐4782. 27078105 10.1073/pnas.1603907113 PMC4855599

[11] Singh S , Vrishni S , Singh BK , Rahman I , Kakkar P . Nrf2‐ARE stress response mechanism: a control point in oxidative stress‐mediated dysfunctions and chronic inflammatory diseases. Free Radic Res. 2010;44:1267‐1288. 20815789 10.3109/10715762.2010.507670

[12] Linker RA , Lee DH , Ryan S , et al. Fumaric acid esters exert neuroprotective effects in neuroinflammation via activation of the Nrf2 antioxidant pathway. Brain. 2011;134:678‐692. 21354971 10.1093/brain/awq386

[13] Nguyen T , Sherratt PJ , Nioi P , Yang CS , Pickett CB . Nrf2 controls constitutive and inducible expression of ARE‐driven genes through a dynamic pathway involving nucleocytoplasmic shuttling by Keap1. J Biol Chem. 2005;280:32485‐32492. 16000310 10.1074/jbc.M503074200

[14] Brennan MS , Matos MF , Li B , et al. Dimethyl fumarate and monoethyl fumarate exhibit differential effects on KEAP1, NRF2 activation, and glutathione depletion in vitro. PLOS One. 2015;10:e0120254. 25793262 10.1371/journal.pone.0120254 PMC4368598

[15] Kensler TW , Wakabayash N , Biswal S . Cell survival responses to environmental stresses via the Keap1‐Nrf2‐ARE pathway. Annu Rev Pharmacol. 2007;47:89‐116. 10.1146/annurev.pharmtox.46.120604.141046 16968214

[16] Ghoreschi K , Bruck J , Kellerer C , et al. Fumarates improve psoriasis and multiple sclerosis by inducing type II dendritic cells. J Exp Med. 2011;208:2291‐2303. 21987655 10.1084/jem.20100977 PMC3201195

[17] Thimmulappa RK , Lee H , Rangasamy T , et al. Nrf2 is a critical regulator of the innate immune response and survival during experimental sepsis. J Clin Invest. 2006;116:984‐995. 16585964 10.1172/JCI25790 PMC1421348

[18] Altmeyer P , Hartwig R , Matthes U . Efficacy and safety profile of fumaric acid esters in oral long‐term therapy of severe psoriasis vulgaris. An investigation of 83 patients. Hautarzt. 1996;47:190‐196. 8647701 10.1007/s001050050401

[19] Rosenkranz T , Novas M , Terborg C . PML in a patient with lymphocytopenia treated with dimethyl fumarate. N Engl J Med. 2015;372:1476‐1478. 25853765 10.1056/NEJMc1415408

[20] Lehmann‐Horn K , Penkert H , Grein P , et al. PML during dimethyl fumarate treatment of multiple sclerosis: how does lymphopenia matter? Neurology. 2016;87:440‐441. 27343070 10.1212/WNL.0000000000002900

[21] Schimrigk S , Brune N , Hellwig K , et al. Oral fumaric acid esters for the treatment of active multiple sclerosis: an open‐label, baseline‐controlled pilot study. Eur J Neurol. 2006;13:604‐610. 16796584 10.1111/j.1468-1331.2006.01292.x

[22] Confavreux C , Vukusic S . Non‐specific immunosuppressants in the treatment of multiple sclerosis. Clin Neurol Neurosurg. 2004;106:263‐269. 15177781 10.1016/j.clineuro.2004.02.012

[23] Lambrecht BN , Hammad H . Taking our breath away: dendritic cells in the pathogenesis of asthma. Nat Rev Immunol. 2003;3:994‐1003. 14647481 10.1038/nri1249

[24] Lambrecht BN , De Veerman M , Coyle AJ , Gutierrez‐Ramos JC , Theilemans K , Pauwels RA . Myeloid dendritic cells induce Th2 responses to inhaled antigen, leading to eosinophilic airway inflammation. J Clin Invest. 2000;106:551‐559. 10953030 10.1172/JCI8107 PMC380243

[25] Gill MA . The role of dendritic cells in asthma. J Allergy Clin Immun. 2012;129:889‐901. 22464668 10.1016/j.jaci.2012.02.028

[26] van Rijt LS , Jung S , KleinJan A , et al. In vivo depletion of lung CD11c(+) dendritic cells during allergen challenge abrogates the characteristic features of asthma. J Exp Med. 2005;201:981‐991. 15781587 10.1084/jem.20042311 PMC2213109

[27] Zaynagetdinov R , Sherrill TP , Kendall PL , et al. Identification of myeloid cell subsets in murine lungs using flow cytometry. Am J Resp Cell Mol. 2013;49:180‐189. 10.1165/rcmb.2012-0366MA PMC3824033 23492192

[28] Jenkins SJ , Perona‐Wright G , Worsley AGF , Ishii N , MacDonald AS . Dendritic cell expression of OX40 ligand acts as a costimulatory, not polarizing, signal for optimal th2 priming and memory induction in vivo. J Immunol. 2007;179:3515‐3523. 17785785 10.4049/jimmunol.179.6.3515

[29] Furuhashi K , Suda T , Hasegawa H , et al. Mouse lung CD103+ and CD11bhigh dendritic cells preferentially induce distinct CD4+ T‐cell responses. Am J Respir Cell Mol Biol. 2012;46:165‐172. 21908266 10.1165/rcmb.2011-0070OC

[30] del Rio ML , Rodriguez‐Barbosa JI , Kremmer E , Forster R . CD103– and CD103+ bronchial lymph node dendritic cells are specialized in presenting and cross‐presenting innocuous antigen to CD4+ and CD8+ T cells. J Immunol. 2007;178:6861‐6866. 17513734 10.4049/jimmunol.178.11.6861

[31] Plantinga M , Guilliams M , Vanheerswynghels M , et al. Conventional and monocyte‐derived CD11b(+) dendritic cells initiate and maintain T helper 2 cell‐mediated immunity to house dust mite allergen. Immunity. 2013;38:322‐335. 23352232 10.1016/j.immuni.2012.10.016

[32] Medoff BD , Seung E , Hong S , et al. CD11b(+) myeloid cells are the key mediators of Th2 cell homing into the airway in allergic inflammation. J Immunol. 2009;182:623‐635. 19109196 10.4049/jimmunol.182.1.623 PMC2718444

[33] Zhu K , Mrowietz U . Enhancement of antibacterial superoxide‐anion generation in human monocytes by fumaric acid esters. Arch Dermatol Res. 2005;297:170‐176. 16187092 10.1007/s00403-005-0598-0

[34] Zhu K , Mrowietz U . Inhibition of dendritic cell differentiation by fumaric acid esters. J Invest Dermatol. 2001;116:203‐208. 11179994 10.1046/j.1523-1747.2001.01159.x

[35] Peng H , Guerau‐de‐Arellano M , Mehta VB , et al. Dimethyl fumarate inhibits dendritic cell maturation via nuclear factor kappaB (NF‐kappaB) and extracellular signal‐regulated kinase 1 and 2 (ERK1/2) and mitogen stress‐activated kinase 1 (MSK1) signaling. J Biol Chem. 2012;287:28017‐28026. 22733812 10.1074/jbc.M112.383380 PMC3431702

[36] Ghoreschi K , Thomas P , Breit S , et al. Interleukin‐4 therapy of psoriasis induces Th2 responses and improves human autoimmune disease. Nat Med. 2003;9:40‐46. 12461524 10.1038/nm804

[37] Litjens NHR , Nibbering PH , Barrois AJ , et al. Beneficial effects of fumarate therapy in psoriasis vulgaris patients coincide with downregulation of type 1 cytokines. Brit J Dermatol. 2003;148:444‐451. 12653735 10.1046/j.1365-2133.2003.05153.x

[38] Gillard GO , Collette B , Anderson J , et al. DMF, but not other fumarates, inhibits NF‐kappa B activity in vitro in an Nrf2‐independent manner. J Neuroimmunol. 2015;283:74‐85. 26004161 10.1016/j.jneuroim.2015.04.006

[39] Spencer SR , Wilczak CA , Talalay P . Induction of glutathione transferases and Nad(P)H‐quinone reductase by fumaric‐acid derivatives in rodent cells and tissues. Cancer Res. 1990;50:7871‐7875. 2123743

[40] Idzko M , Hammad H , van Nimwegen M , et al. Local application of FTY720 to the lung abrogates experimental asthma by altering dendritic cell function. J Clin Invest. 2006;116:2935‐2944. 17080194 10.1172/JCI28295 PMC1626118

[41] Sawicka E , Zuany‐Amorim C , Manlius C , et al. Inhibition of Th1‐ and Th2‐mediated airway inflammation by the sphingosine 1‐phosphate receptor agonist FTY720. J Immunol. 2003;171:6206‐6214. 14634137 10.4049/jimmunol.171.11.6206

[42] Vermaelen KY , Carro‐Muino I , Lambrecht BN , Pauwels RA . Specific migratory dendritic cells rapidly transport antigen from the airways to the thoracic lymph nodes. J Exp Med. 2001;193:51‐60. 11136820 10.1084/jem.193.1.51 PMC2195883

[43] Rubant SA , Ludwig RJ , Diehl S , et al. Dimethylfumarate reduces leukocyte rolling in vivo through modulation of adhesion molecule expression. J Invest Dermatol. 2008;128:326‐331. 17671516 10.1038/sj.jid.5700996

[44] Chen H , Assmann JC , Krenz A , et al. Hydroxycarboxylic acid receptor 2 mediates dimethyl fumarate's protective effect in EAE. J Clin Invest. 2014;124:2188‐2192. 24691444 10.1172/JCI72151 PMC4001545

[45] Tang H , Lu JYL , Zheng XM , Yang YH , Reagan JD . The psoriasis drug monomethylfumarate is a potent nicotinic acid receptor agonist. Biochem Bioph Res Co. 2008;375:562‐565. 10.1016/j.bbrc.2008.08.041 18722346

[46] Vander Lugt B , Khan AA , Hackney JA , et al. Transcriptional programming of dendritic cells for enhanced MHC class II antigen presentation. Nat Immunol. 2014;15:161‐167. 24362890 10.1038/ni.2795

[47] Figdor CG , van Kooyk Y , Adema GJ . C‐type lectin receptors on dendritic cells and Langerhans cells. Nat Rev Immunol. 2002;2:77‐84. 11910898 10.1038/nri723

[48] Clarke DL , Davis NHE , Campion CL , et al. Dectin‐2 sensing of house dust mite is critical for the initiation of airway inflammation. Mucosal Immunol. 2014;7:558‐567. 24129160 10.1038/mi.2013.74 PMC3998635

[49] Ito T , Hirose K , Norimoto A , et al. Dectin‐1 plays an important role in house dust mite‐induced allergic airway inflammation through the activation of CD11b( + ) dendritic cells. J Immunol. 2017;198:61‐70. 27852745 10.4049/jimmunol.1502393

[50] Jember AGH , Zuberi R , Liu FT , Croft M . Development of allergic inflammation in a murine model of asthma is dependent on the costimulatory receptor OX40. J Exp Med. 2001;193:387‐392. 11157058 10.1084/jem.193.3.387 PMC2195923

