# Supplementary figures and images for: Dimethyl fumarate abrogates dust mite‐induced allergic asthma by altering dendritic cell function
Source: Immun Inflamm Dis. 2019 Jul 2;7(3):201–13. doi: 10.1002/iid3.262 (PMC6688084; doi:10.1002/iid3.262)

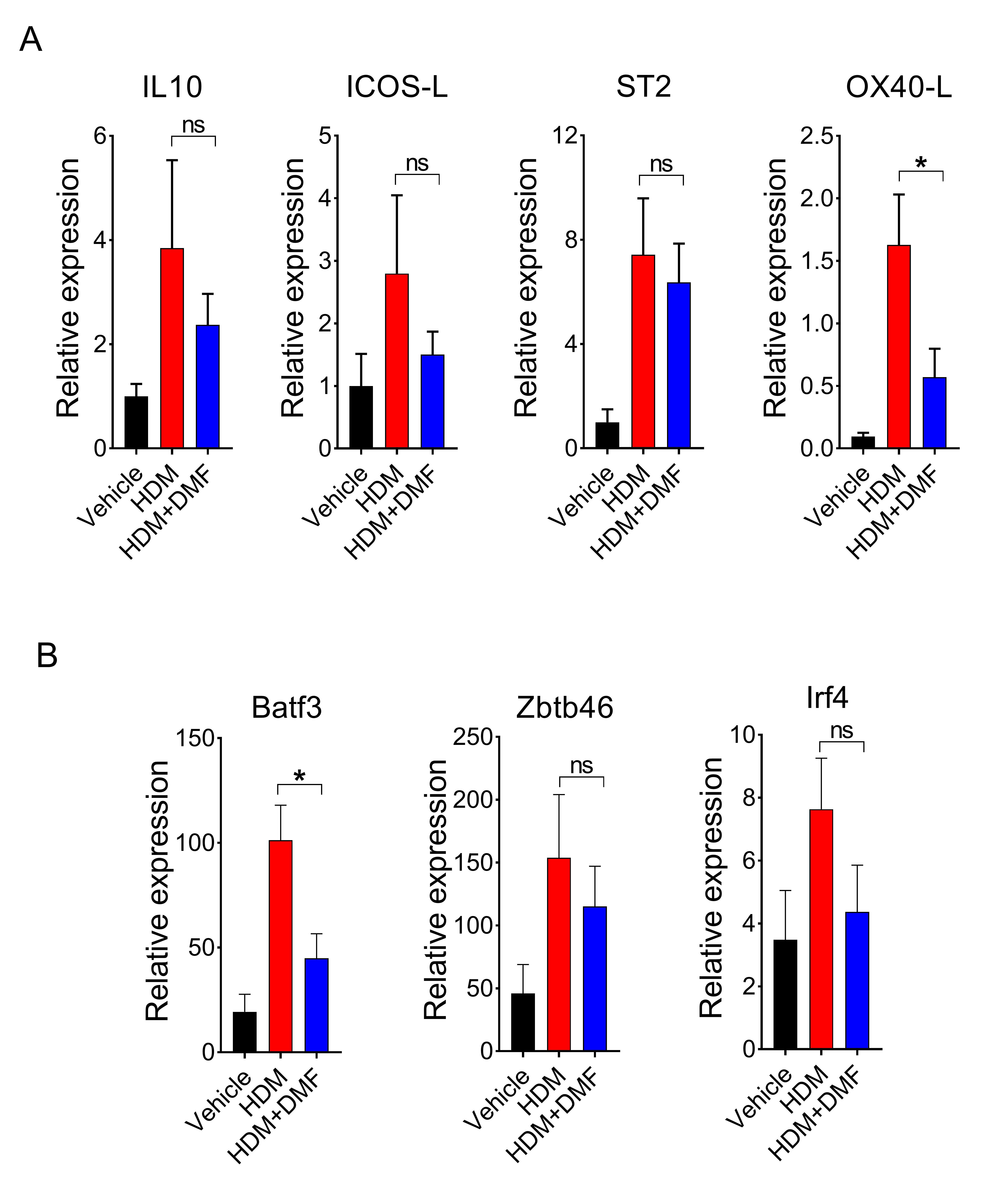

Supplement: Supplementary file 1 — Supplementary Figure 1. Effect of DMF treatment on costimulatory and transcription factor gene expressions in DCs. Splenic DCs were pulsed with HDM (100 µg ml−1) and were cultured overnight in presence or absence of DMF (75 µM). DCs‐specific relative expression of A) HDM‐driven immune pathway and B) transcription factor genes are shown. Results are pooled from at least 2 independent experiments and values represent means ± SEMs. (n = 4‐6, *P < 0.05, or P = ns, unpaired t‐test, HDM versus HDM+DMF‐treated DCs) [file IID3-7-201-s001.jpg]

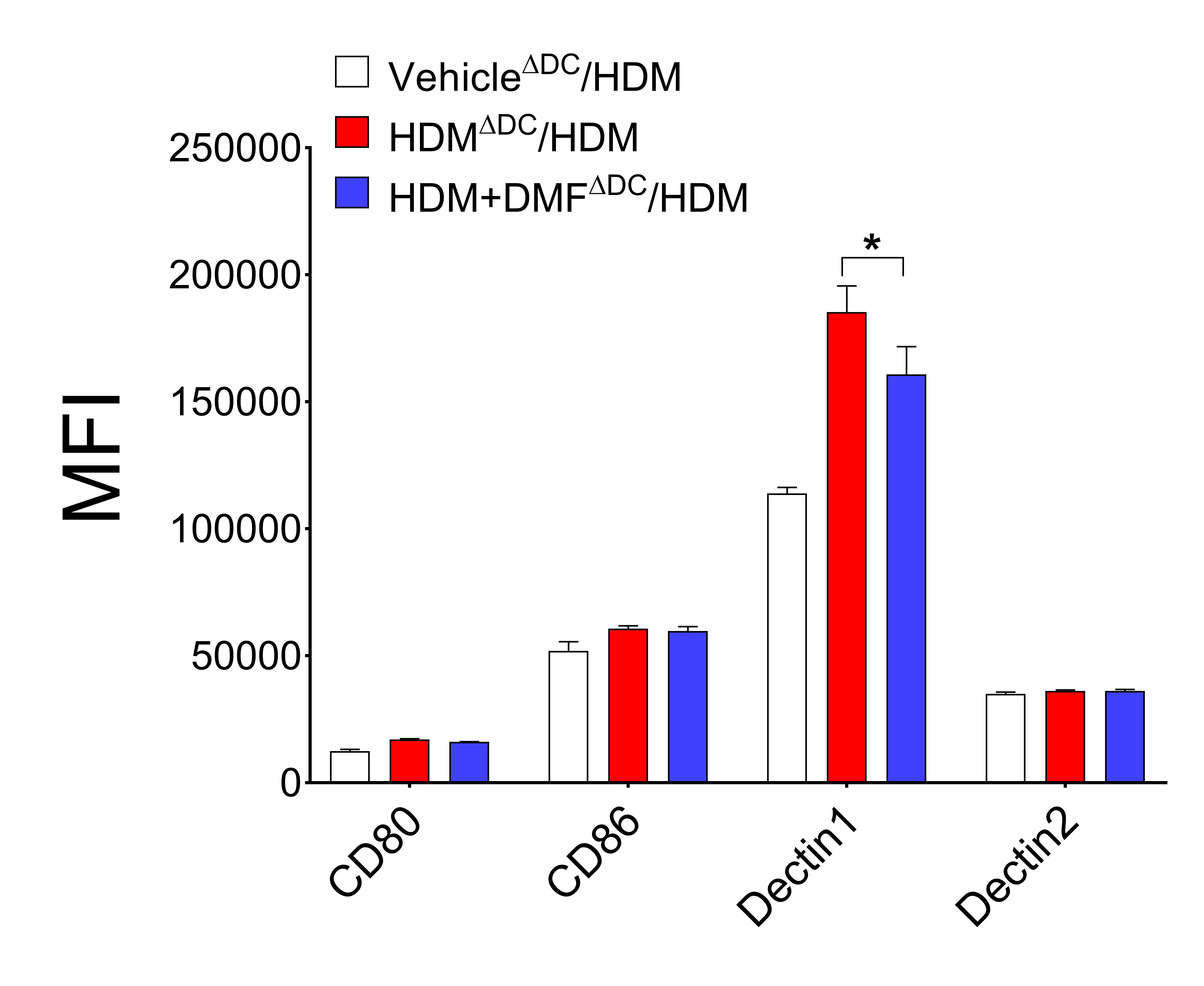

Supplement: Supplementary file 2 — Supplementary Figure 2. Activation profile of lung DCs from recipient mice sensitized with DMF treated BMDCs differs from untreated mice. Cell surface expression of mean fluorescence intensity (MFI) of A) CD80 B) CD86 C) Dectin‐1, and D) Dectin‐2 by lung SiglecF /SSClo / CD11c+ / MHCII hi DCs from recipient mice that received HDM‐pulsed BMDCs either DMF‐treated or untreated and challenged with HDM. Results are representative of data generated in two different experiments (n= 4 mice /group) [file IID3-7-201-s002.jpg]

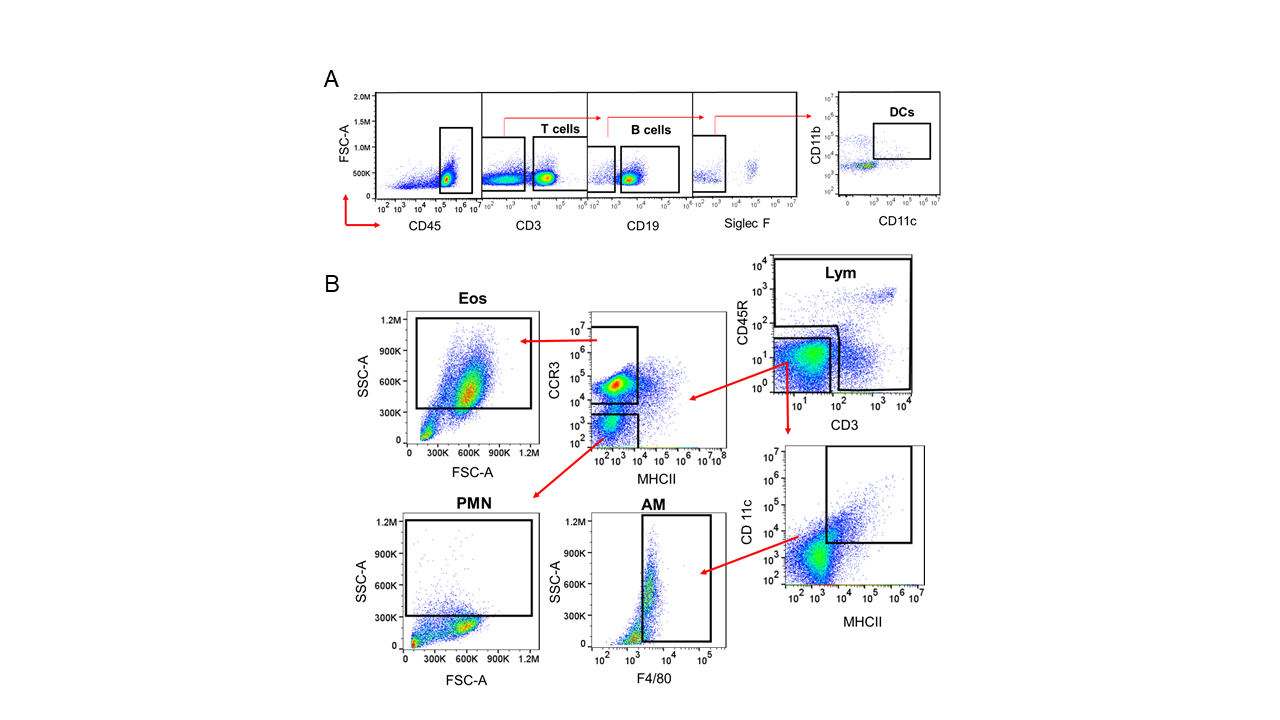

Supplement: Supplementary file 3 — Supplementary Figure 3. Gating strategy for enumeration of immune cells in lymph nodes and BALF sample. CD11b+ DC were identified in mLNs and pLNs samples as SiglecF‐/CD11c+/CD11b+ (A), and (B) differential counting of BALF cells were analyzed by flow cytometry using sequential gating analysis. Lymphocytes (Lym) were identified as CD3+/ CD45R+/ MHCII‐ cells, and the CD3‐ / CD45R‐ cell population were gated as CD11c+/ MHCII+/ F4/80+ alveolar macrophages (AM); F4/80‐/ SSC hi/ CCR3‐ neutrophil (PMN); and SSC hi/ MHC II‐/ CCR3+ eosinophil (Eos) [file IID3-7-201-s003.tif]
